# The Surgical Treatment of Gallstones and Inflammations of the Biliary Tract1Read before the Buffalo Academy of Medicine, Section on Surgery, April 6, 1909.

**Published:** 1910-01

**Authors:** Martin B. Tinker

**Affiliations:** Ithaca, N. Y.; Assistant Professor of Surgery, Cornell University


### The Surgical Treatment of Gallstones and Inflammations of the Biliary Tract[1]

By MARTIN B. TINKER, S. B., M. D., Ithaca, N. Y.
Assistant Professor of Surgery, Cornell University

THE operative treatment of the biliary tract disorders has been so frequently and so ably discussed in recent years that relatively little remains to be said that is not entirely familiar to all interested in this subject. Hence, I must beg your indulgence with this paper, which I have endeavored to make as brief as possible and fairly cover the ground.

In opening a discussion on this subject, a word in honor of the memory of John Stough Bobbs, of Indianapolis, seems fitting. Doubtless, many of you have read about Bobb's pioneer cholecystostomy. A few years ago, I went over the literature on this subject (*Johns Hopkins Hospital Bulletin,* Vol. XII, August, 1901), and I feel sure that any of you who will review the evidence, will feel convinced that to America belongs the credit of the first surgical intervention for gallstones as well as for the first ovariotomy, nephrectomy, and appendectomy. I should be glad to devote more time to history of the surgery of the gall passages, which seems to me quite as interesting and profitable a

1. Read before the Buffalo Academy of Medicine, Section on Surgery, April 6, 1909.

subject for study as the history of political intrigues and injustices and of the slaughter of war. I judge, however, that the clinical side of the question chiefly concerns us tonight.

In the forty-two years since Bobbs first drained the gall-bladder, much progress has been made. We now deal successfully with the severer forms of inflammation and stone in the deeper bile passages; conditions which would have probably been considered beyond all possibility of surgical relief, even up to 1880 after Marion Sims as well as Bobbs had successfully drained the gall-bladder. Topics which seem of general interest touching our present operative treatment of the bile passage are:

1. What factors have contributed to the safety of our surgery and the permanence of cure?
2. What results may be expected from surgical treatment?
3. How can the results of surgical treatment be improved?

### SEVERE INFLAMMATIONS.

The methods of dealing with acute, severe inflammations; with stone; and with gall tract conditions associated with neighboring organs differ widely. It is true that inflammation and stone are usually associated; indeed in the light of our present knowledge, gallstones probably result, as a rule, from infection, but in speaking of inflammation, I refer to those cases of acute inflammation progressing rapidly; going on to distension of the gall-bladder with pus, frequently to gangrene and perforation. In most cases such rapidly progressive inflammations supervene after the formation of stone; in a smaller number the inflammation is the first symptom. In these cases I believe the best results are obtained by methods diametrically opposed to those giving the best results in operating for stone alone. Speed is of great importance. We should do as little as possible, drain freely, usually protecting our field with a large gauze pack, instead of the small cigaret or split tube drainage which we use in ordinary cases. As has been said: "Get in, but above all get out as soon as possible." Drainage with the use of local anesthesia is frequently sufficient to give relief and save life. A later secondary operation can be done for stone if necessary, after the acute symptoms have subsided and recovery is assured. This get in, get out, and drain freely policy has greatly improved results in treating inflammation.

### STONE IN THE DUCTS.

In operating for stone, however, especially stone in the deeper ducts, care and thoroughness count for much. Among improvements in technic worthy of note are, the rendering the deeper bile passages more accessible by elevation of the dorsal spine over a thick roll as suggested by Mayo Robson, which brings the duodenum at the junction with the common bile duct several inches nearer to the anterior abdominal wall, gravitates the intestines away from the field of operation, and greatly facilitates operation upon the deeper bile passages. A second factor of even greater importance is the thorough protection of the remainder of the abdominal cavity from soiling by the use of abundant gauze packing. Our walling off with gauze is now far more thorough than when I began my surgical training fifteen years ago. The bile is always infected in these cases and the packing should be sufficiently abundant so that bile does not escape from the immediate neighborhood of the field of operation causing peritonitis, which in former times with less care was frequently fatal.

A third factor, early introduced, is the drainage of the kidney pouch, into which the bile tends to collect if there is not provision for free drainage, in case there is leakage from the ducts after operation. A stab wound in the groin was formerly used, but I believe a carefully placed split rubber tube with gauze can be carried from the operative wound with less mutilation of the patient, and with equally satisfactory results. The split tube with gauze not only takes care of any ordinary amount of leakage, but provides a path of lessened resistance for the escape of fluid in case later leakage occurs. A fourth factor which has doubtless saved many lives, is the routine use of drainage of the gall-bladder or ducts in these cases.

Seven years ago, in a paper presented before the Surgical Section of the American Medical Association at Saratoga, based upon twenty-seven cases of common duct stone operations, in the Johns Hopkins Hospital up to that time (*American Medicine,* 1902, Vol. III, Page 1039), I advocated routine drainage of the bile passages. At that time this suggestion was disapproved by a number of prominent surgeons present. Since that time the confidence in the value of routine drainage has, I believe, become almost universal. If drainage tubes be carefully fastened in place by catgut sutures and surrounded by a moderate amount of gauze, leakage of bile is unusual and the healing of the wound is almost as rapid and solid as per priam, as a rule. In cholecystectomy. I usually leave a stump of cystic duct and drain from that. This allows free flow of bile to the intestines. Kehr, (*Zentralblatt für Chirurgie,* 1909, No. 1, page 3), has recognised the importance of not entirely obstructing the flow of bile into the intestines by inserting a drain into the hepatic duct as he formerly advised, and as has been practised by most German surgeons. To obviate this he now uses a T-tube. When the cystic duct is not strictured, drainage directly from it seems a simpler method.

### THE INCISION.

The incision in an operation for stone should be ample to give room for thorough exploration of the ducts. For several years I have used and advocated muscle splitting and retraction in opening the abdomen. At a meeting of the Chicago Medical Society in November, 1907, (*Illinois Medical Journal*, January, 1905), I described a muscle retraction method for general use in abdominal surgery, which I had then used for three years and which is almost identical with the incision described by Clifford, of Peoria, Ill., in a recent number of *Surgery, Gynecology, and Obstetrics*. It has the advantage of giving abundant room for exposure of the ducts, minimum injury to nerves and aponeurotic layers, ready closure, and no danger of ventral hernia or abdominal weakness.

### EXPLORATION OF THE DUCTS.

In all uncomplicated operations for stone, a probe should be passed through the ducts into the duodenum giving assurance that the common duct is free from obstruction. If there is difficulty in this, further search should be made for the cause of the obstruction which should be removed before we are satisfied to close the wound. If the incision is adequate in size and position, the patient properly placed on the table with the spine at the duodenum elevated over a roll, the intestines properly packed away with gauze pads and suitable retraction used, the ducts can almost always be readily examined by sight and palpation. With such care, I believe that a stone will seldom, if ever, be overlooked. That stones occasionally form in the smallest branches of the hepatic duct which may later give rise to obstruction and trouble, has been conclusively shown by the experience of McArthur (*Surgery, Gynecology, and Obstetrics*, 1905, Vol. I., Page 158), who demonstrated the presence of such stones post mortem. But I judge that such cases are extremely rare.

### LOCAL ANESTHESIA.

In case the patient has organic disease of the heart, lungs, kidneys, or other important organ or in case of feebleness or advanced old age, I would urge the use of local anesthesia which in my opinion greatly reduces the risk of operation in such cases. Cholecystostomy can be performed with entire satisfaction under local anesthesia. Cholecystectomy will probably require the use of a few drams of ether or chloroform for a painless operation. I would not have you think that I advise local anesthesia except in cases where general anesthesia would be attended by unusual risk; in such cases my colleagues and I are convinced that its use frequently means the difference between life and death. Four patients with whom I have used local anesthesia were all much weakened by prolonged infection and pain; two were partly convalescent with typhoid when gall-bladder infection developed; the worst risk was a woman of seventy with stones, stricture of the cystic duct, and bad valvular lesion of the heart. All recovered. Lennander of Upsala, Sweden; Mitchell, of Washington, and others have reported similar experiences.

### GALLSTONES AND APPENDICITIS.

The pathological conditions associated with the biliary tract affections will surely be dealt with fully by Dr. Park, in the discussion on the complications. Additional emphasis on the importance of two frequently associated conditions, however, will do no serious harm, I refer to appendicitis and movable kidney. The frequent association of appendicitis and biliary tract inflammation has been mentioned by several writers. Unless this association is kept in mind and the appendix is removed in proper cases, our patients will fail to get relief from gall-bladder pain in about 10 per cent. of the cases. Removal of the appendix through the same incision used to expose the biliary tract, I have always found possible where necessary and usually easy.

### GALL-DUCT OBSTRUCTION AND MOVABLE KIDNEY.

Before the Surgical Section of the American Medical Association two years ago, I reported two cases of gall-duct obstruction dependent upon movable kidney.—(*Journal American Medical Association,* July 13, 1907). The association of biliary tract affections with movable kidney has received but little notice in American medical literature, in spite of great activity in abdominal surgery in this country in recent years. The condition is, I believe, not especially rare or unusual, but of relative frequency. In contrast to the infrequent mention of gall-tract affections in association with movable kidney, gastric and intestinal indigestion have been frequently mentioned in this connection.

The relation between certain cases of appendicitis and movable kidney has been emphasized by Edebohls and others and appropriate surgical treatment recommended. That extreme renal morbility should cause appendicitis in some cases, gastric disturbance in others, and not affect the near neighbors of those organs, the gall-bladder and biliary ducts is hardly conceivable. That such obstruction actually occurs, I have had the opportunity to observe in three cases. Very little has appeared in the literature on this subject. Cordier has reported six cases in which gallstones were associated with movable kidney due, he believes, to compression of the common duct. Von Tischendorf performed nephrorrhaphy in one case on the peritoneal side after an operation for biliary calculus with a successful result. Apolant believes that there are many cases at Carlsbad whose symptoms are due to movable kidney, but are wrongly attributed to biliary tract lesions. It has been my endeavor not to exaggerate the frequency or overestimate the importance of gall-tract obstruction from movable kidney. It is certain that the subject has seldom been mentioned in the literature and that such cases have been overlooked on the operating table, occasionally at least. If we are always to give our patients the degree of relief which they confidently expect when they consent to abdominal operation, we must recognise and correct all causes of trouble within the abdomen, whether of frequent or infrequent occurrence.

RESULTS SHOULD EQUAL THOSE OF OPERATION FOR APPENDICITIS.

As to the immediate results of operative treatment, much depends on the patient's condition. Mayo Robson, Moynihan, and Kehr tell us that in simple uncomplicated cases, mortality should be less than 1 per cent. I see no reason why the risk should not be much less than 1 per cent. in such cases, operated upon by an experienced, competent surgeon under favorable conditions. It surely should not be greater than for uncomplicated appendicitis. I have yet to see the first death in an uncomplicated case, either in my own practice or in that of those with whom I have been associated. Among the complicated cases, it is a wonder that as many recover as do. During the past five years, I have lost two patients: one a woman who had been jaundiced from stone impacted in the common duct for over six months. She had taken little food during that time, and had lost enormously in weight and strength. Death resulted apparently from lack of strength to repair, a week after operation. There was no evidence of infection or other failure in operative methods.

The second patient, a woman of seventy, had had severe attacks of gallstone colic, frequently with jaundice, for over twenty-five years; three large stones were removed from the common duct and five from the gall-bladder. There was quite marked cirrhosis of the liver. This patient died two weeks after operation, probably as a result of cholemia from cirrhosis. These are the only fatalities out of a series of cases that include three gangrenous gall-bladders, two perforations, two cases of suppurative cholecystitis after a prolonged siege of typhoid fever, two patients over seventy years of age, seven patients having complications not directly concerning the biliary tract, such as valvular disease of the heart, advanced arteriosclerosis, chronic nephritis, and other complicating conditions.

### TYPHOID CHOLECYSTITIS.

As an example of the desperate cases that may recover, I would briefly report the history of a woman living in Ithaca, who came under my care two years after the famous typhoid fever epidemic. She had not been well following a severe attack of fever. She suffered from constant indigestion and was able to take only the simplest foods, often only liquids. Dyspepsia, in this case, was an important sign of the trouble, but associated with it in these infections, pain is almost always present, and this particular case was no exception. There was almost constant dull aching pain in the upper right quadrant of the abdomen. A mass appeared and as the patient had lost over forty pounds in weight, malignant disease was suspected by her physician. The pain was never extremely severe. The symptoms of indigestion were constant, there was never a very high fever; jaundice was not present. In this state of ill health and suffering, she managed to exist for two years. At that time the gall-bladder had become adherent to the abdominal wall. Pus had burrowed through the abdominal wall and there was a fluctuating mass beneath the skin. The patient's condition was so bad that I felt that only drainage was advisable.

Later, she went from under my care, returning after several months with another extensive abscess that needed opening. Since then this woman has gained fifty pounds in weight and is now in perfect health, with the exception of the fact that she has a mucus fistula, caused probably by closing of the cystic duct during the inflammation. This would be curable by removal of the gall-bladder, which the patient has not yet consented to allow me to undertake. I might also add that we saved some of the pus from the abscess and were able to grow in the laboratory, a pure culture of the typhoid bacillus, although two years after the attack of fever. There were three or four small stones which I got, but it was the typhoid pus that was causing the chief part of the trouble.

### ULTIMATE RESULTS.

As to the permanency of results, much also depends upon the condition of the patient at the time of operation, whether complete or palliative operation is advisable; whether the irritation and prolonged inflammation has caused permanent disorder of the digestive functions, cirrhosis of the liver, pancreatitis, and extensive adhesions; or whether the condition is taken early. Much, too, depends upon the thoroughness of the work of the surgeon. If the bile tracts have been thoroughly explored, after the general plan outlined, I believe that recurrence of symptoms of stone will be the very rarest exception.

WHEN SHOULD SURGICAL TREATMENT BE ADVISED?

No topic in connection with biliary tract surgery seems to me of greater consequence. As with many another condition, delays are more dangerous than surgery. Judging from my own experience the profession as a whole, advises surgery far less early than for appendicitis, or indeed, for many other diseases of equal or less gravity. On looking over my case histories with this point of delay in mind, I find that over one-third of the patients have suffered many years. Among the series are noted symptoms of twenty years duration; of eighteen years (a physician too); twelve years, eleven years, two of eight years, six years, and many of three or four years duration. Several of these patients were referred by physicians who would, undoubtedly, have advised operation for acute appendicitis within twelve hours of the onset of the attack. All doubtful cases should surely be given the benefit of thorough trial of medical measures, but three to twenty years seems unnecessarily long to test the merits of olive oil, phosphate, succinate and salicylate of soda and Carlsbad water.

Simply because a patient has escaped the immediate danger of operation, does not imply that he has escaped other dangers equally great or possibly greater. As with acute appendicitis, the attacks of pain usually recur. Probably the inflammatory conditions of the biliary tract are not quite as dangerous to life as inflammation of the appendix, and I would not go so far as some of my surgical friends who would treat gallstones as they would pus,—wherever present advise evacuation. However, in view of the fact that these affections generally give rise to repeated attacks of atrocious pain, that lives have been frequently and unnecessarily sacrificed from gangrene, perforation and general peritonitis; from prolonged jaundice, and from such complications as acute hemorrhagic pancreatitis; that there is definite evidence that prolonged irritation of gallstones is among the most frequent causes of chronic indigestion, often erroneously supposed by the patient to be stomach trouble; in view of these facts and of many others which I have not mentioned, should not operative intervention be advised earlier than is usual with the average physician? And to this end, that there shall be earlier operation in needful cases, education of the public as well as the rank and file of the profession is needed, better trained surgeons are needed and coöperation between physicians and surgeons is needed.

Frequent indiscriminating advice for operation is certain to bring ultimate discredit to the surgeon. On the other hand over conservatism and failure to see the surgical aspects of these cases does not bring credit to the physician. We see certain aspects of disease that you do not; you are familiar with much that would puzzle us. A careful study of doubtful cases by the attending physician and the consulting surgeon working together is certain to be mutually helpful, and for the best good of the patient.

